# Identify metabolism-related genes IDO1, ALDH2, NCOA2, SLC7A5, SLC3A2, LDHB, and HPRT1 as potential prognostic markers and correlate with immune infiltrates in head and neck squamous cell carcinoma

**DOI:** 10.3389/fimmu.2022.955614

**Published:** 2022-08-25

**Authors:** Ce Li, Shuai Chen, Wenming Jia, Wenming Li, Dongmin Wei, Shengda Cao, Ye Qian, Rui Guan, Heng Liu, Dapeng Lei

**Affiliations:** Department of Otorhinolaryngology, Qilu Hospital of Shandong University, National Health Commission (NHC) Key Laboratory of Otorhinolaryngology (Shandong University), Jinan, China

**Keywords:** hypopharyngeal squamous cell carcinoma, metabolic reprogramming, cellular stress, immune response, tumor infiltrating lymphocytes

## Abstract

Hypopharyngeal squamous cell carcinoma (HSCC) is a kind of head and neck squamous cell carcinoma (HNSCC) with poor prognosis. Metabolic reprogramming may regulate the tumor microenvironment (TME) by adapting quickly to cellular stress and regulating immune response, but its role in HSCC has not been reported. We used the nCounter^®^ Metabolic Pathways Panel to investigate metabolic reprogramming, cellular stress, and their relationship in HSCC tissues and adjacent normal tissues. Metabolism-related pathways nucleotide synthesis and glycolysis pathways were significantly upregulated, while amino acid synthesis and fatty acid oxidation pathways were significantly downregulated in HSCC tissues compared to adjacent normal tissues. There is a significant correlation between metabolism-related pathways and cellular stress pathways. Enrichment of immune cell and tumor infiltrating lymphocyte (TIL) analysis showed changes in immune responses between HSCC tissues and adjacent normal tissues. Overall survival analysis showed that upregulated genes CD276, LDHB, SLC3A2, EGFR, SLC7A5, and HPRT1 are potential unfavorable prognostic markers in HNSCC, while downregulated genes EEA1, IDO1, NCOA2, REST, CCL19, and ALDH2 are potential favorable prognostic markers in HNSCC. Moreover, metabolism-related genes IDO1, ALDH2, NCOA2, SLC7A5, SLC3A2, LDHB, and HPRT1 are correlated with immune infiltrates in HNSCC. These results suggest that metabolic reprogramming occurs and correlates with cellular stress and immune response in HSCC, which may help researchers understand mechanisms of metabolic reprogramming and develop effective immunotherapeutic strategies in HNSCC.

## Introduction

Hypopharyngeal carcinoma is a kind of head and neck tumor which occurs between the level of the hyoid bone and the lower end of the cricoid cartilage (1). The most common form of hypopharyngeal cancer is squamous cell carcinoma (SCC), which has the worst prognosis of all head and neck malignancies (2). Although hypopharyngeal carcinoma accounts for only 3%–5% of head and neck malignancies, the 5-year survival rate is only 30% to 35% (3, 4). Hypopharyngeal cancer usually presents at an advanced stage and requires aggressive treatment, including surgery, chemotherapy, and radiation therapy; however, the survival rate did not improve significantly (5, 6).

Abnormal energy metabolism is one of the important features of tumor cells. In the 1930s, Otto Warburg found that tumor cells prefer glycolysis, and even in the condition of sufficient oxygen supply, tumor cells still maintain vigorous glycolysis and consume a lot of glucose. This abnormal metabolism is called “Warburg effect” (7). Although the genetic theory of carcinogenesis is widely accepted, research in recent years confirms Warburg’s earlier observations that aerobic glycolysis plays an important role in tumorigenesis. For example, large-scale metabolic profiling finds that rapidly proliferating tumor cells are highly dependent on glycine (8). Lactic acid can directly participate in the tricarboxylic acid cycle as an energy substrate, combined with the function of helping cells escape immune damage, and effectively promote the growth and invasion of tumor cells under hypoxic conditions (9, 10). Tumor cell lipid metabolism can also undergo reprogramming, affecting basic life processes such as signal transduction and gene expression and promoting tumor progression (11). Recent studies have shown that abnormal metabolite or intermediate metabolism in cancer may regulate immune cell proliferation, differentiation, activation, and function (12, 13). Metabolic reprogramming has been studied in a range of tumors, including lung cancer (14), liver cancer (15), and glioma (16). However, metabolic reprogramming and immune response in HSCC have not been researched.

Cells are regularly exposed to potentially life-threatening stressful environments, including DNA damage, oxidative stress, starvation, endoplasmic reticulum (ER) stress, and hypoxia (17). Tumor cells proliferate rapidly by changing the way of energy metabolism to quickly adapt to the cellular stress microenvironment (18). Cellular stress can modulate the cross talk between immune cells and tumor cells, remodeling tumor immunogenicity, immune function, and phenotype (19). Moreover, the correlation between metabolic reprogramming and cellular stress in HSCC has not been researched.

In this study, we studied metabolic reprogramming and cellular stress and their relationships in HSCC by using NanoString nCounter^®^ Metabolic Pathways Panel. Metabolism and cell stress-related genes and pathways in HSCC and adjacent normal tissues were detected and analyzed. We also analyzed the expression levels of different types of immune cells and tumor-infiltrating lymphocytes (TILs) in HSCC tissues and adjacent normal tissues. Moreover, correlations of altered genes with tumor were analyzed in KEGG cancer pathways, and expression levels and overall survival time of altered genes were analyzed in TCGA HNSCC database. The gene correlation with immune infiltrates was also analyzed. In brief, we analyzed metabolic reprogramming, cellular stress, and immune response in HSCC and identified genes, prognostic markers, and signaling pathways that play roles in HNSCC.

## Methods

### Clinical sample preparation

Twelve pairs of HSCC tissues and adjacent normal tissues were obtained from HSCC patients and stored at -20°C. All patient data used for study were approved by the Ethics Committee of Qilu Hospital of Shandong University. Patients participating in the program were informed. Human ethical approval was from the Ethics Committee of Qilu Hospital of Shandong University.

### nCounter^®^ metabolic pathways panel

The nCounter Metabolic Pathways Panel v1.0 was used to quantify the expression of genes involved in core metabolic processes and immune metabolism in human samples, including Biosynthesis and Anabolic Pathways theme, Cell Stress theme, Nutrient Capture and Catabolic Pathways theme, Metabolic Signaling theme, and Transcriptional Regulation theme and 768 genes. The presence and relative abundance of 14 different immune cell types for immunometabolism studies were quantified (**Table S1**). Details are available in https://nanostring.com/products/ncounter-assays-panels/oncology/metabolic-pathways/.

### Sample loading for nCounter^®^ metabolic pathways panel

A cartridge containing a hybridization reaction for each sample was set up using the following components: 3 μl Reporter CodeSet, 5 μl Hybridization Buffer, 5 μl (50 ng) sample RNA, and 2 μl Capture ProbeSet at room temperature. The cartridge was incubated in a preheated 65°C PCR machine, and hybridization was performed for at least 16 h. After hybridization, 15 µl RNase-free water was mixed well, and the cartridge was put into the instrument and operated on the NanoString nCounter platform.

### Metabolic pathways panel analysis

nSolver 4.0 software for the NanoString platform was used for analyses. Gene expression of one HSCC tissue was inseparable from the background because of sample degradation; this sample was excluded from the analysis. Finally, 11 HSCC tissues and 12 adjacent normal tissues were included in the analysis.

### Gene expression normalization

Normalization is determined by calculating the normalization factor of the sample according to housekeeper genes, then multiplying the raw data by the normalization factor to get normalized data. Housekeeper genes EDC3, POLR2A, COG7, SDHA, NRDE2, FCF1, AGK, MRPS5, DHX16, DNAJC14, TBC1D10B, SAP130, TLK2, STK11IP, and TBP were used for normalization.

### Pathway scoring module

Pathway scoring was used to aggregate data from pathway genes into a single score. At least one covariate must be selected to plot the scores, while the effects of other variables that may be highly correlated with gene expression can be removed from the analysis by adjusting the scores for these variables. Pathway scores were calculated as the first principal component of the normalized expression of pathway genes.

### Cell type profiling module

The method described by Danaher (2017) was used to measure the abundance of various cell populations (20). This method quantifies cell populations using marker genes that are stably and specifically expressed in a given cell type, which serve as reference genes specific to individual cell types.

### PathView module

The PathView module overlays differential expression analysis results with various KEGG pathways. Elements that are overexpressed in this pathway are golden, those that are underexpressed are blue, and those that are unchanged are gray.

### Analysis of dysregulated gene expression in TCGA HNSCC database

Gene expression analysis of HNSCC patients in The Cancer Genome Atlas (TCGA) database was performed by using GEPIA (http://gepia.cancer-pku.cn/ (21).

### Analysis of correlation between overall survival and gene expression in TCGA HNSCC database

Correlation analysis between gene expression and overall survival time of HNSCC patients in TCGA database was performed by using GEPIA (http://gepia.cancer-pku.cn/ (21).

### Immune infiltration analysis

Correlations between prognostic gene expression levels and immune infiltrations were performed by using TIMER (https://cistrome.shinyapps.io/timer/ (22, 23).

### Statistical analysis

Experimental data were shown as mean ± standard deviation (SD). Results of different groups were compared using a two-tailed Student’s t-test. Differences were considered statistically significant when the P value was less than 0.05.

## Results

### Analysis of differentially expressed metabolism-related genes in HSCC

**Figure 1:**
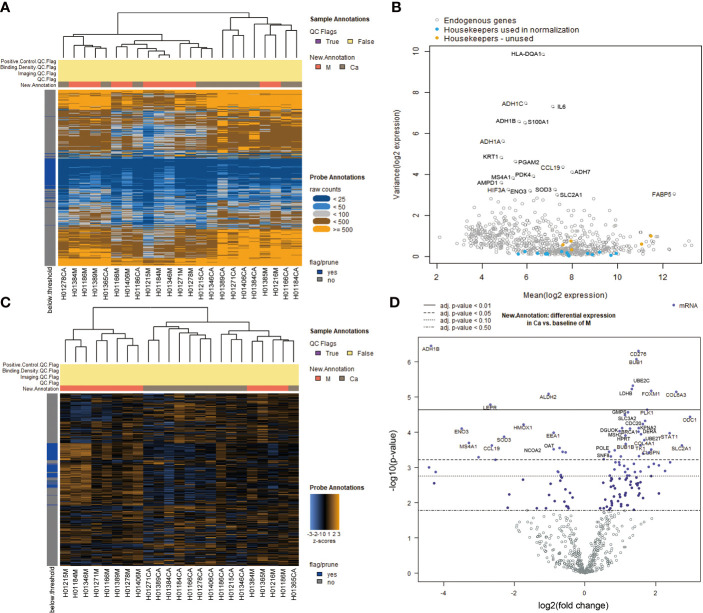
Analysis of differentially expressed genes in Metabolic Pathways Panel between HSCC tissues and adjacent normal tissues.**(A)** Heatmap of the raw counts. The plot is meant to provide an overview of how robust the raw expression levels are across samples and gene sets. **(B)** Variance vs. mean normalized signal plot across all targets/probes. Each gene’s variance in the log-scaled, normalized data is plotted against its mean value across all samples. Highly variable genes are indicated by gene name. Housekeeping genes are color coded according to their use in (or omission from) normalization. **(C)** Heatmap of the normalized data, scaled to give all genes equal variance, generated *via* unsupervised clustering. Orange indicates high expression; blue indicates low expression. This plot is meant to provide a high level exploratory view of the data. **(D)** Volcano plot: New.Annotation: Ca vs. M volcano plot displaying each gene’s -log10 (p-value) and log2 fold change with the selected covariate. Highly statistically significant genes fall at the top of the plot above the horizontal lines, and highly differentially expressed genes fall to either side. Horizontal lines indicate various false discovery rate (FDR) thresholds or p-value thresholds if there is no adjustment to the p-values. Genes are colored if the resulting p-value is below the given FDR or p-value threshold. The 40 most statistically significant genes are labeled in the plot. (Ca: HSCC tissues M: adjacent normal tissues).

We studied the expression levels of metabolism-related genes in 12 pairs of hypopharyngeal carcinoma and adjunct normal tissues by using NanoString nCounter^®^ Metabolic Pathways Panel. A heatmap of the raw counts provided an overview of the raw expression levels of gene sets in samples (**Figure 1A**). A volcano plot showed changes of endogenous genes and housekeeping genes between HSCC tissues and adjacent normal tissues (**Figure 1B**). Raw expression levels of genes were normalized by using part of housekeeping genes (**Table S2, S3**). A heatmap of the normalized data provided an overview of the global expression changes of gene sets in HSCC tissues and adjacent normal samples (**Figure 1C**). A volcano plot showed significantly differentially expressed genes between HSCC tissues and adjacent normal tissues (**Figure 1D**). ODC1, COL6A3, FOXM1, PLK1, and CDC20 were the most significant upregulated metabolism-related genes, while ADH1B, ENO3, LEPR, and ALDH2 were the most significant downregulated metabolism-related genes in HSCC tissues compared with adjacent normal tissues (**Table 1**).

**Table 1:** Analysis of genes with the most significant difference in nCounter^®^ Metabolic Pathways Panel between HSCC and adjacent normal tissues.

Gene name	Log2 fold change	P-value	Pathway
ADH1B-mRNA	-4.36	3.64E-07	Amino acid synthesis, fatty acid oxidation, glycolysis
CD276-mRNA	1.52	4.97E-07	TCR and costimulatory signaling
BUB1-mRNA	1.46	8.55E-07	Cell cycle
UBE2C-mRNA	1.35	4.93E-06	Antigen presentation, cell cycle
LDHB-mRNA	1.34	6.01E-06	Amino acid synthesis, glycolysis, mitochondrial respiration
FOXM1-mRNA	1.88	6.68E-06	Cell cycle, transcriptional regulation
COL6A3-mRNA	2.59	7.33E-06	PI3K
ALDH2-mRNA	-1.03	8.30E-06	Amino acid synthesis, fatty acid oxidation, glycolysis, tryptophan kynurenine metabolism
LEPR-mRNA	-2.68	1.65E-05	AMPK, cytokine and chemokine signaling
PLK1-mRNA	1.76	2.34E-05	Cell cycle
GMPS-mRNA	1.22	2.72E-05	Nucleotide synthesis
SLC3A2-mRNA	1.15	3.27E-05	Amino acid transporters, mTOR
ODC1-mRNA	2.97	3.73E-05	Amino acid synthesis, Myc
CDC20-mRNA	1.7	4.93E-05	Antigen presentation, cell cycle
KPNA2-mRNA	1.64	5.87E-05	Cytokine and chemokine signaling, DNA damage repair
HMOX1-mRNA	-1.74	6.10E-05	Cytokine and chemokine signaling, KEAP1NRF2 pathway, reactive oxygen response
DERA-mRNA	1.51	7.73E-05	Pentose phosphate pathway
DGUOK-mRNA	1.05	7.84E-05	Nucleotide salvage, nucleotide synthesis
ENO3-mRNA	-3.49	8.06E-05	Glycolysis
CTPS1-mRNA	1.28	8.15E-05	Nucleotide synthesis

### Analysis of altered metabolism-related pathways in HSCC

**Figure 2:**
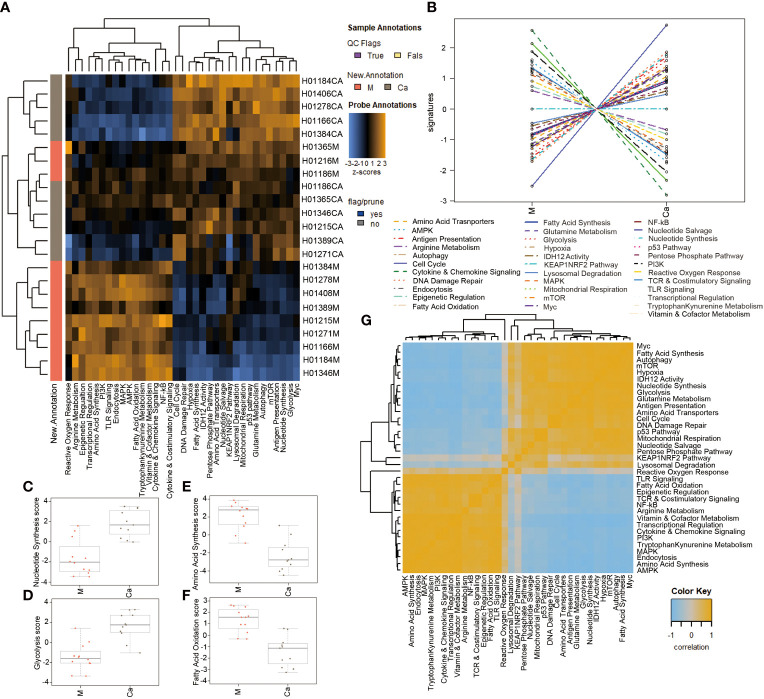
Analysis of differential signaling pathways in Metabolic Pathways Panel between HSCC tissues and adjacent normal tissues.**(A)** Heatmap showing the pathway scores. This plot is a high-level overview of how the pathway scores change across samples. Orange indicates high scores; blue indicates low scores. **(B)** Heatmap showing the correlation matrix of pathway scores. Orange and blue indicate positive and negative correlation, respectively. **(C, D)** Pathway scores of significantly upregulated pathway in HSCC tissues. **(E, F)** Pathway scores of significantly downregulated pathways in HSCC tissues. **(G)** Heatmap of the correlation matrix of pathway scores. Heatmap showing the correlation matrix of pathway scores. Orange and blue indicate positive and negative correlation, respectively. (Ca: HSCC tissues M: Adjacent normal tissues).

A heatmap of pathway scores provided an overview of how the pathway scores change across samples (**Figure 2A**). A line chart of pathway scores showed the altered metabolism and cellular stress-related pathways in HSCC (**Figure 2B**). Cell cycle, DNA damage repair, nucleotide synthesis, antigen presentation, glycolysis, and mTOR pathway were significantly upregulated in HSCC tissues compared with normal tissues (**Figure S1**). Cytokine and chemokine, amino acid synthesis, PI3K, transcriptional regulation, AMPK, and TLR signaling pathway were significantly downregulated in HSCC tissues compared with normal tissues (**Figure S2**). For metabolism-related signaling pathways, nucleotide synthesis and glycolysis pathways were significantly upregulated (**Figures 2C, D**), while amino acid synthesis and fatty acid oxidation pathways were significantly downregulated in HSCC tissues compared to adjacent normal tissues (**Figures 2E, F**). A heatmap of the correlation matrix of pathway scores showed that there is a significant correlation between metabolism-related pathways and cellular stress-related pathways (**Figure 2G**). These results suggest that metabolic reprogramming correlates with cellular stress in HSCC.

### Expression and correlation of different types of immune cells in HSCC and adjacent normal tissues

**Figure 3:**
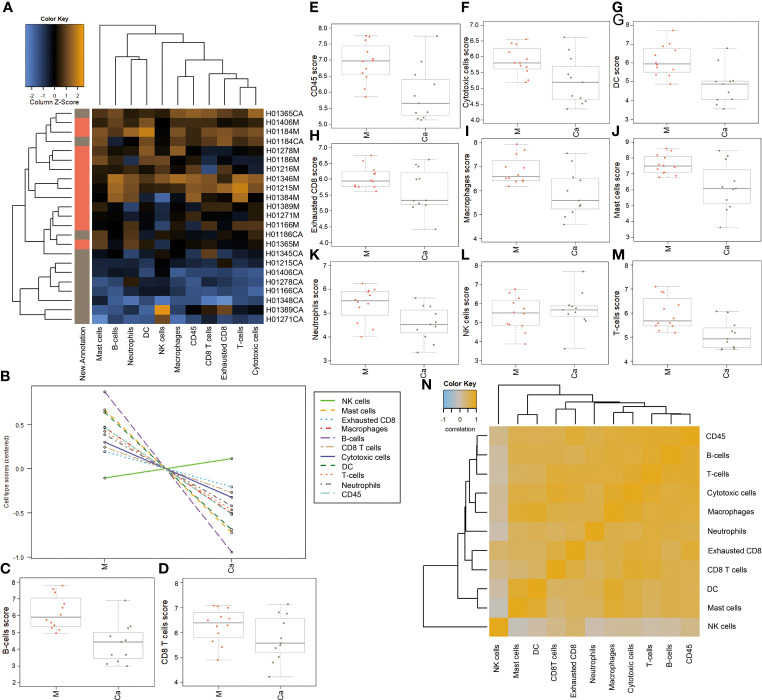
Abundance of immune cells in HSCC tissues and adjacent normal tissues.**(A)** Heatmap of raw cell type measurements. Orange indicates high abundance; blue indicates low abundance **(B)** Raw cell type measurements vs. New.Annotation. plots the raw cell type abundance measurements. **(C)** Plots B-cell measurements. **(D)** Plots CD8 cells measurements. **(E)** Plots CD45 measurements. **(F)** Plots cytotoxic cell measurements. **(G)** Plots DC measurements. **(H)** Plots exhausted CD8-cell measurements. **(I)** Plots macrophage measurements. **(J)** Plots mast-cell measurements. **(K)** Plots neutrophil measurements. **(L)** Plots NK-cell measurements. **(M)** Plots T-cell measurements. **(N)** Heatmap of the correlation matrix of raw cell type measurements. Heatmap showing the correlation matrix of raw cell abundance. Orange and blue indicate positive and negative correlations, respectively. Ordinate: log_2_TPM (Ca: HSCC tissues M: Adjacent normal tissues).

We analyzed the expression levels of different types of immune cells in HSCC tissues and adjacent normal tissues. A heatmap of raw cell type measurements provided an overview of how the immune cell types change across samples (**Figure 3A**). A line chart of raw cell type measurements showed that the abundance of NK cells was increased, while the abundance of mast cells, exhausted CD8, macrophages, B cells, CD8 T cells, cytotoxic cells, DC, T cells, neutrophils, and CD45 was decreased in HSCC tissues compared with normal tissues (**Figure 3B**). Scatter diagrams showed measurements of the above cells in each HSCC tissue and normal tissue (**Figures 3C–M)**. A heatmap of the correlation matrix of raw cell type measurements showed correlations between different types of immune cells (**Figure 3N**). There is a significant positive correlation between mast cells, macrophages, and DC.

**Figure 4:**
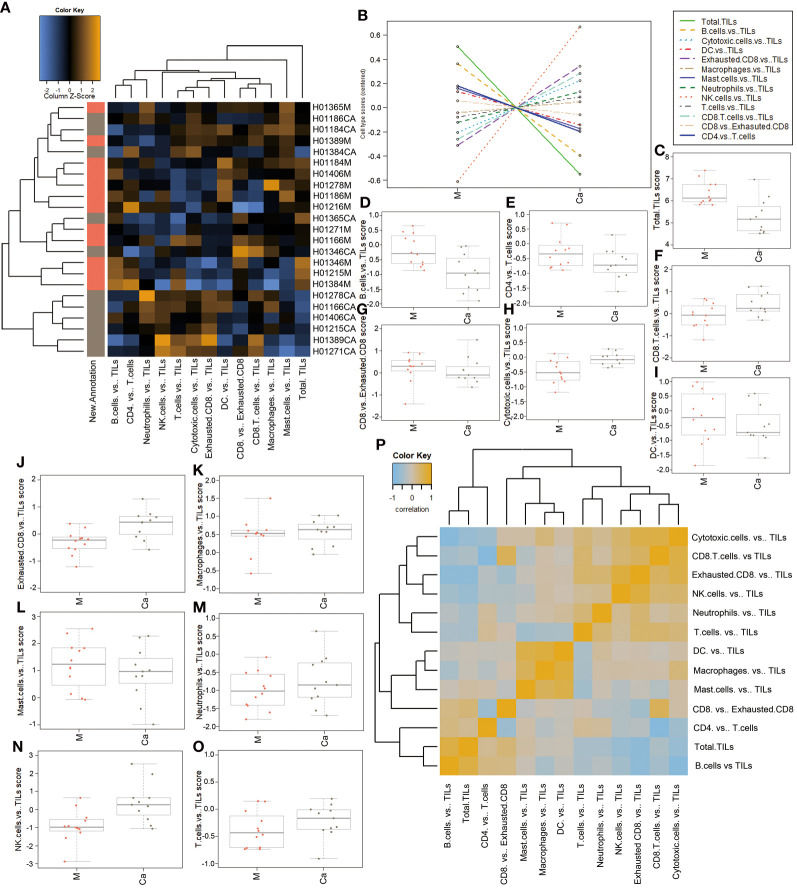
Correlation between immune response cells and immune infiltration in HSCC tissues and adjacent normal tissues.**(A)** Heatmap of relative cell type measurements. Orange indicates high abundance; blue indicates low abundance. **(B)** Relative cell type measurements vs. New.Annotation plots the relative cell type abundance measurements. **(C)** Each panel plots total TILs against another relative abundance measurements. Points are colored by New.Annotation. **(D)** Each panel plots B cells vs. TILs against another relative abundance measurements. Points are colored by New.Annotation. **(E)** Each panel plots CD4 cells vs. T cells against another relative abundance measurements. Points are colored by New.Annotation. **(F)** Each panel plots CD8 cells vs. TILs against another relative abundance measurements. Points are colored by New.Annotation. **(G)** Each panel plots CD8 vs. exhausted CD8 against another relative abundance measurements. Points are colored by New.Annotation. **(H)** Each panel plots cytotoxic cells vs. TILs against another relative abundance measurement. Points are colored by New.Annotation. **(I)** Each panel plots DC vs. TILs against another relative abundance measurements. Points are colored by New.Annotation. **(J)** Each panel plots exhausted CD8 vs. TILs against another relative abundance measurements. Points are colored by New.Annotation. **(K)** Each panel plots macrophages vs. TILs against another relative abundance measurements. Points are colored by New.Annotation. **(L)** Each panel plots mast cells vs. TILs against another relative abundance measurements. Points are colored by New.Annotation. **(M)** Each panel plots neutrophils vs. TILs against another relative abundance measurements. Points are colored by New.Annotation. **(N)** Each panel plots NK cells vs. TILs against another relative abundance measurements. Points are colored by New.Annotation. **(O)** Each panel plots T cells vs. TILs against another relative abundance measurements. Points are colored by New.Annotation. **(P)** Heatmap of the correlation matrix of relative cell type measurements. Heatmap showing the correlation matrix of relative cell abundance. Orange and blue indicate positive and negative correlations, respectively. Ordinate: log_2_TPM (Ca: HSCC tissues M: adjacent normal tissues).

We also analyzed the expression levels of tumor-infiltrating lymphocytes (TILs) in HSCC tissues and normal tissues, which included B cells, T cells, cytotoxic cells, and macrophages. A heatmap of raw cell type measurements provided an overview of how the immune cell types against Tils change across samples (**Figure 4A**). A line chart of raw cell type measurements against Tils showed that cytotoxic cells vs. TILs, exhausted CD8 vs. TILs, cytotoxic cells vs. TILs, NK cells vs. TILs, T cells vs. TILs, and CD8 T cells vs. TILs were increased, while total TILs, B cells vs. TILs, DC vs. TILs, mast cells vs. TILs, CD8 vs. exhausted CD8, and CD4 vs. T cells were decreased in HSCC tissues compared with normal tissues (**Figure 4B**). Scatter diagrams showed measurements of the above immune cells vs. TILs in HSCC tissues and normal tissues (**Figures 4C–O**). A heatmap of the correlation matrix of raw cell type measurements showed correlations between different types of immune cells vs. TILs (**Figure 4P**). There is a significant positive correlation between mast cells vs. Tils, macrophages vs. Tils, and DC vs. Tils. Our results illuminated changes of abundance of different types of immune cells, immune infiltrations, and their relationship in HSCC and normal hypopharyngeal tissue.

### Analysis of altered genes in KEGG cancer pathways

**Figure 5:**
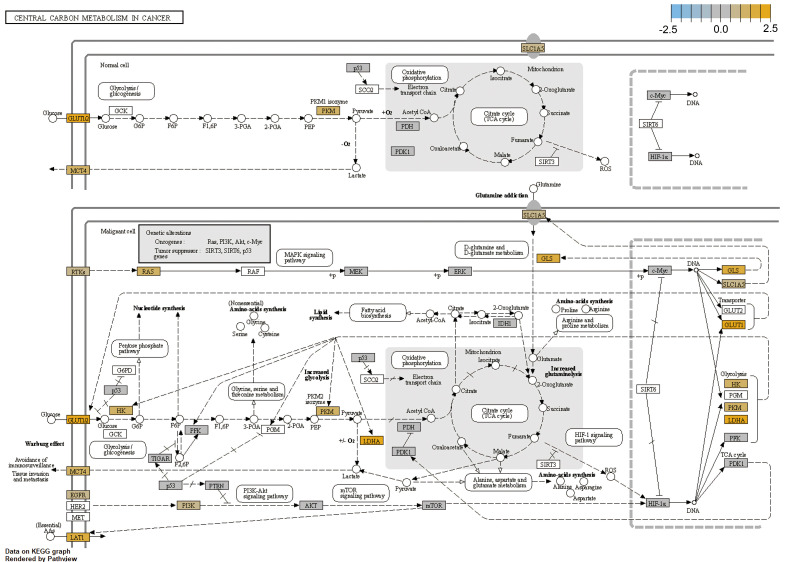
Analysis of altered gene in HSCC in cancer carbon metabolism pathways.For the cancer carbon metabolism KEGG pathway, genes within the panel were mapped to pathways, and differential expression information was overlaid on protein-based KEGG pathway images.

We then analyzed altered genes in HSCC in KEGG cancer carbon metabolism pathways and cancer pathways. In cancer carbon metabolism pathways, the Warburg effect obviously appeared in HSCC; glycolysis-related gene HK, PKM, and LDHA were upregulated; and transporter genes GLS, SLC1A5, MCT4 and GLUT1 were upregulated (**Figure 5**). In the cancer pathway, cell-cycle gene cyclin A1 and cyclin D1 were upregulated, while tumor proliferation and metastasis pathway FAK-PI3K and Ras-PI3K were upregulated; DNA damage repair-related gene RAD51 was also upregulated. These results revealed potential pathways and mechanisms of proliferation, metastasis, and radiation resistance in HSCC (**Figure S3**). RAS, PI3K, and EGFR, which are involved in both glycolysis pathways and cancer pathway, were significantly upregulated. These results may provide evidence of the mutual promoted effects for tumor pathway and metabolism pathway.

### Analysis of expression levels of altered metabolism-related genes in HSCC and correlation with overall survivals in TCGA HNSCC database

**Figure 6:**
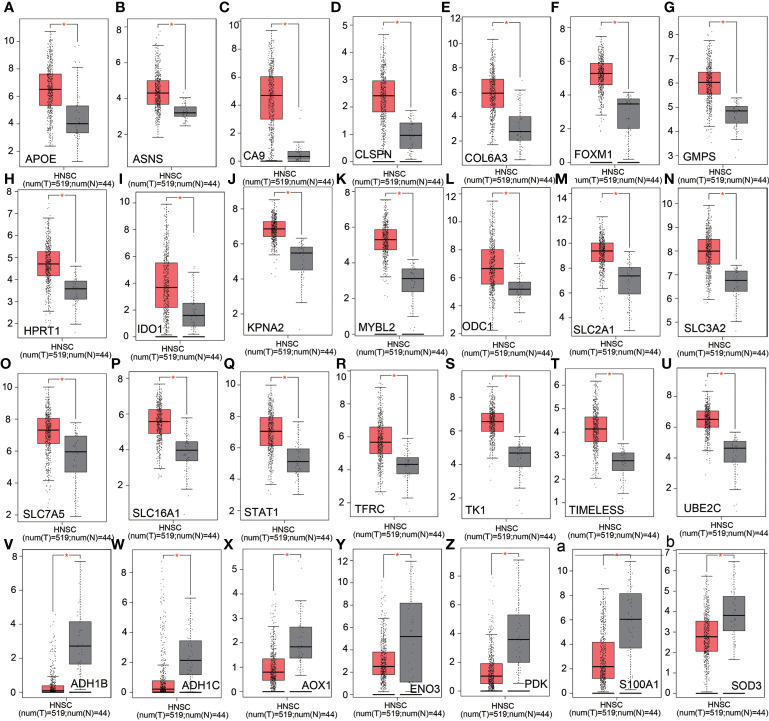
Analysis of relative expression level of altered genes in nCounter^®^ Metabolic Pathways Panel in TCGA HNSCC database. **(A–U)** Relative expression levels of upregulated genes in Metabolic Pathways Panel in TCGA HNSCC database. **(V–Z)** and **(a, b)** Relative expression levels of downregulated genes in Metabolic Pathways Panel in TCGA HNSCC database. Red: HNSCC tissues. Gray: adjacent normal tissues. Ordinate: log_2_ (TPM+1).

**Figure 7:**
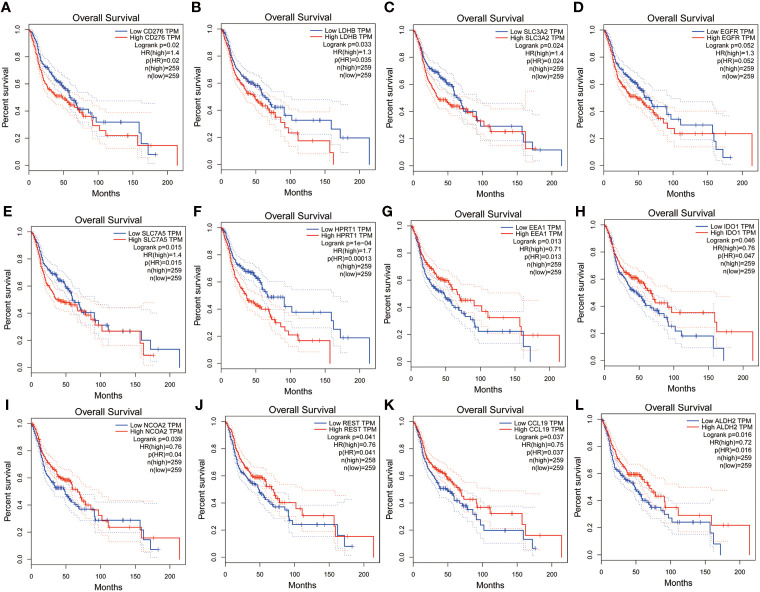
Analysis of overall survival of altered genes in nCounter^®^ Metabolic Pathways Panel in TCGA HNSCC database.**(A-F)** Analysis of altered genes in Metabolic Pathways Panel with unfavorable prognosis in TCGA HNSCC database. **(G-L)** Analysis of genes in Metabolic Pathways Panel with favorable prognosis in TCGA HNSCC database.

We analyzed the expression levels of altered metabolism-related genes in HSCC in TCGA HNSCC database. APOE, ASNS, CA9, CLSPN, COL6A3, FOXM1, GMPS, HPRT1, IDO1, KPNA2, MYBL2, ODC1, SLC2A1, SLC3A2, SLC7A5, SCLC16A1, STAT1, TFRC, TK1, TIMELESS, and UBE2C were significantly upregulated in HNSCC tissues compared with normal tissues (**Figures 6A–U**). On the other hand, ADH1B, ADH1C, AOX1, ENO3, PDK, S100A1, and SOD3 were significantly downregulated in HNSCC tissues compared with normal tissues (**Figures 6V–Z, a, b**). Overall survival analysis in TCGA HNSCC database showed that CD276, LDHB, SLC3A2, EGFR, SLC7A5, and HPRT1 were potential unfavorable prognosis markers in HNSCC (**Figures 7A–F**), while EEA1, IDO1, NCOA2, REST, CCL19, and ALDH2 were potential favorable prognosis markers in HNSCC (**Figures 7G–L**). Among these prognosis-related genes, LDHB, SLC3A2, SLC7A5, HPRT1, IDO1, NCOA2, and ALDH2 are metabolism-related genes, while EEA1, CD276, EGFR, REST, and CCL19 are cellular stress-related genes. These results suggest that metabolism-related genes correlate with prognosis in HNSCC.

### Analysis of relationships between prognosis-related genes and immune infiltrates in HNSCC

**Figure 8:**
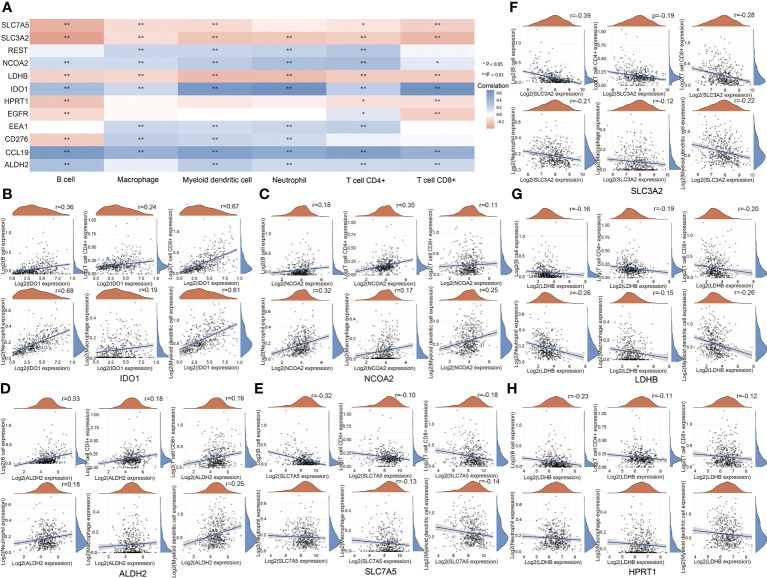
Analysis of relationships between prognosis-related genes and immune infiltrates.**(A)** Relationships between prognosis-related genes and immune infiltrates in HNSCC. **(B)** Relationships between IDO1 and immune infiltrates in HNSCC. **(C)** Relationships between NCOA2 and immune infiltrates in HNSCC. **(D)** Relationships between ALDH2 and immune infiltrates in HNSCC. 9 **(E)** Relationships between SLC7A5 and immune infiltrates in HNSCC. **(F)** Relationships between SLC3A2 and immune infiltrates in HNSCC. **(G)** Relationships between LDHB and immune infiltrates. **(H)** Relationships between HPRT1 and immune infiltrates in HNSCC. (*P < 0.05; **P < 0.01).

At last, we studied the relationships between prognosis-related genes and immune infiltrates in HNSCC. IDO1, REST, NCOA2, EEA1, CLL19, and ALDH2 showed a positive correlation with immune infiltrates, while SLC7A5, SLC3A2, LDHB, and HPRT1 showed a negative correlation with immune infiltrates in the HNSCC database (**Figure 8A**). For metabolism-related genes, there is a positive correlation between IDO1 and NCOA2 expression levels and B cells, CD4+ T cells, CD8+ T cells, neutrophils, macrophages, and myeloid dendritic cells (**Figures 8B, C**). There is a positive correlation between ALDH2 expression level and B cells, CD4+ T cells, CD8+ T cells, neutrophils, and myeloid dendritic cells (**Figure 8D**). On the other hand, there is a negative correlation between SLC7A5 expression level and B cells, CD4+ T cells, CD8+ T cells, macrophages, and myeloid dendritic cells (**Figure 8E**). There is a negative correlation between SLC3A2 and LDHB expression levels and B cells, CD4+ T cells, CD8+ T cells, neutrophils, macrophages, and myeloid dendritic cells (**Figures 8F, G**). There is a negative correlation between HPRT1 expression level and B cells, CD4+ T cells, and CD8+ T cells (**Figure 8H**). These results suggest that metabolic reprogramming correlates with immune response in HNSCC.

## Discussion

Head and neck tumors are the sixth most common malignant tumors worldwide. In 2018, there were nearly 800,000 new cases and about 400,000 deaths worldwide. The annual overall survival rate was lower than 50% (24). Most head and neck cancers arise from the mucosal epithelial cells of the mouth, pharynx, and larynx, collectively referred to as head and neck squamous cell carcinomas (HNSCCs) (25). Hypopharyngeal squamous cell carcinoma (HSCC) remains one of the most lethal malignancies of HNSCC (26). Discovering new targets in HSCC is of great significance for the clinical treatment and diagnosis of hypopharyngeal carcinoma.

In this study, we found that nucleotide synthesis and glycolysis pathways were significantly upregulated, while amino acid synthesis and fatty acid oxidation pathways were significantly downregulated in HSCC. NK cells were upregulated while other types of immune cells and total TILs were downregulated in HSCC. KEGG pathway analysis showed that glycolysis-related genes HK, PKM, and LDHA as well as transporter genes GLS, SLC1A5, MCT4, and GLUT1 were upregulated in HSCC. RAS, PI3K, and EGFR, which are involved in both glycolysis pathways and cancer pathway, were significantly upregulated in HSCC. Moreover, metabolism-related genes IDO1, ALDH2, NCOA2, SLC7A5, SLC3A2, LDHB, and HPRT1 were identified as potential prognostic markers and correlate with immune infiltrates in HNSCC. Overall, our results confirm that metabolic reprogramming occurs and correlates with cellular stress and immune response in HSCC, which may help researchers understand mechanisms of metabolic reprogramming and develop effective immunotherapeutic strategies in HNSCC.

The immune function includes the body’s recognition and removal of foreign invading antigens and mutated or senescent cells in the body, as well as maintenance of the stability of the body’s internal environment. It is divided into three aspects according to the different objects of action. Immune response refers to the process by which the immune system recognizes and clears “non-self” substances and is divided into innate immunity and acquired immunity according to the speed and specificity of the response (27). The immune system and malignant cells interact through a complex network. The importance of immune function in tumor development and control has been recognized for decades. Interactions between tumor and immune system include elimination, homeostasis, and escape (28). The tumor microenvironment (TME) is composed of tumor cells, immune cells, tumor-associated fibroblasts, signaling molecules, and extracellular matrix (ECM) (29). Immune cells contains T cells, B cells, dendritic cells (DC), myeloid-derived suppressor cells (MD-SCs), tumor-associated macrophages (TAMs), tumor-associated neutrophils (TANs), and natural killer (NK) cells (30). Lymphocytes infiltrating the tumor local area are called tumor-infiltrating lymphocytes (TILs). The infiltration of CD4+/CD8+ T cells in the tumor stroma is closely related to the prognosis and survival rate of tumor patients. Tumor patients with more tumor stromal lymphocyte infiltration show better overall survival (OS) and tumor-free survival (31). Emerging evidence suggests that cancer cells suppress the antitumor effects of immune cells by competing for or depleting essential nutrients for the immune response as well as reducing the metabolic fitness of tumor-infiltrating immune cells (32). Microenvironmental immunosuppression mediated by metabolic reprogramming is a bottleneck that leads to tumor immune escape and limits the improvement of immunotherapy efficacy (33).

Our results showed that the glycolysis pathway was significantly upregulated in HSCC. For nearly two decades, the Warburg effect has attracted the attention of scientists around the world. Studies have demonstrated that aerobic glycolysis plays an important role in tumorigenesis; for example, isocitrate dehydrogenase (IDH) oncogenic mutations have been found in gliomas and leukemias (34, 35), suggesting that metabolic adjustment may be a central link in cancer. We identify LDHB as an unfavorable prognostic marker which negative correlates with immune infiltrates in HNSCC. Lactate dehydrogenase B (LDHB) can convert lactate to pyruvate, thus promoting oxidative metabolism and producing NADPH (36, 37). LDHB provides sufficient energy for tumor-cell proliferative capacity while avoiding the accumulation of intercellular lactate (38). We identify ALDH2 as a favorable prognostic marker which positively correlates with immune infiltrates in HNSCC. Acetaldehyde dehydrogenase 2 (ALDH2) not only is involved in aldehyde metabolism but also plays a key role in tumor growth. Patients with hepatocellular carcinoma (HCC) with high ALDH2 expression have a good prognosis. ALDH2 is associated with proliferation, metastasis, and multidrug resistance (MDR) of cancer cells (39).

Our results showed that the nucleotide synthesis pathway was significantly upregulated in HSCC. Recent studies have shown that abnormal nucleotide metabolism not only accelerates tumor development but also suppresses normal immune responses in the tumor microenvironment (40). During tumor pathogenesis, under conditions of metabolic stress or hypoxia, tumor and immune cells produce adenosine, which is broken down into precursor purine nucleotide (37). Adenosine receptors (A1, A2A, A2B, and A3) are found on the surface of various immune cells. Increased levels of adenosine in the tumor environment inhibit the lytic activity of natural killer cells through the binding of adenosine to A2A receptors (41). We identify IDO1 as a favorable prognostic marker and positively correlates with immune infiltrates in HNSCC. IDO1 (indoleamine 2,3dioxygenase 1) is a dioxygenase, which is involved in the kynurenine pathway of tryptophan metabolism (42). IDO1 is overexpressed in many kinds of tumor cells, which reduces the level of intracellular tryptophan and the production of a series of metabolites, thus in turn affecting the function of the immune system (43). At the same time, a high expression of IDO1 is also associated with poor prognosis of the disease in many types of tumor patients (44). We identify HPRT1 as an unfavorable prognostic marker and negatively correlates with immune infiltrates in HNSCC. HPRT1 (hypoxanthine phosphoribosyl transferase 1) encodes a key enzyme involved in the purine salvage synthesis pathway. It plays a crucial role in regulating the cell cycle and has been reported to be overexpressed in a variety of cancers (45). The elevated expression of the HPRT1 gene is associated with the progression of HNSCC, which may serve as a useful indicator for early detection, risk stratification, and targeted therapy in HNSCC patients (46).

Our results showed that the amino acid synthesis pathway was significantly downregulated in HSCC. Amino acids are key nutrients for immune cells, and amino acid supply can regulate immune cell function. Immune cells have specific amino acid requirements that induce rapid proliferation by stimulating growth factors and T-cell activation, thereby increasing the expression of amino acid transporters (47, 48). Anabolic or catabolic dysregulation of glutamine, serine, and glycine has been identified as a metabolic regulator that supports cancer cell growth (49). In addition, amino acid derivatives contribute to epigenetic regulation and immune responses associated with tumorigenesis and metastasis (50).

The solute carrier (SLC) family utilizes electrochemical potential differences or ionic gradients generated by major active transporters to transport their substrates across biological membranes. We identify SLC7A5 and SLC3A2 as unfavorable prognostic markers which negatively correlate with immune infiltrates in HNSCC. SLC7A5 and SLC3A2 are important amino acid transporters, which provide key raw amino acids for the growth of cells and organelles and important cellular processes by regulating the transport of amino acids, thereby affecting cell proliferation and differentiation (51, 52). The high expression level of SLC7A5, also named LAT1, is associated with poor prognosis in tumor patients. The pharmacological inhibition or knockdown of SLC7A5 can inhibit the proliferation of cancer cells and the growth of xenograft tumors. SLC7A5 is a candidate molecular target for cancer diagnosis and therapy (53). SLC3A2, also named CD98, is highly expressed in many cancers and is associated with tumor aggressiveness and metastasis. SLC3A2 supports lymphocyte clonal expansion and promotes lymphocyte clonal expansion by promoting integrin signaling proliferation and inhibiting apoptosis for protective adaptive immunity. These integrin-dependent signals can also trigger tumor cell proliferation and invasion (54). Moreover, SLC7A5 and SLC3A2 enhance the metabolic capacity and effector function of tumor-directed CAR-NK and T cells (55).

Our results showed that the fatty acid oxidation pathway was significantly downregulated while the fatty acid synthesis pathway was upregulated in HSCC. Mitochondrial fatty acid beta-oxidation (FAO) is a major source of bioenergy. FAO is dysregulated in a variety of human malignancies. The proliferation, survival, stemness, drug resistance, and metastatic progression of cancer cells depend on FAO. FAO also reprograms in cancer-associated immune cells and other host cells, which may contribute to the immunosuppressive and pro-tumor microenvironment (56). Fatty acid synthesis (FAS) is an important cellular process that converts nutrients into metabolic intermediates for membrane biosynthesis, energy storage, and production of signaling molecules. Cancer cells depend on FAS as a component of cell membrane formation, energy storage, and production of signaling molecules. Targeting multiple points in the metabolic pathway of fatty acid metabolism may disrupt rapid cancer cell proliferation (57). We identify NCOA2 as a favorable prognostic marker which positively correlates with immune infiltrates in HNSCC. NCOA2 (nuclear receptor coactivator 2) is a member of the steroid receptor coactivator family. As a PPARr coactivator, it plays an important role in lipid metabolism and energy balance (58). The NCOA2 gene plays a crucial role in the occurrence, development, and metastasis in many malignant tumors by activating the WNT pathway (59–61).

In this study, we found that cell cycle, DNA damage repair, and mTOR pathway were significantly upregulated in HSCC. There is an unusually complex relationship between cellular stress and cancer immune surveillance. Short-term stress can enhance immune protection or the acquisition of immune pathological responses. Conversely, chronic stress can suppress protective immune responses or exacerbate pathological immune responses (62). Each type of stress typically has different and opposite effects on antitumor immunity which may constitute a pitfall for treating cancer patients with drugs that trigger cellular stress. For example, while DNA-damaging agents, such as many chemotherapeutic drugs used to treat patients with different types of cancer, are able to elicit antitumor immune responses through the upregulation of immunostimulatory stress-regulating molecules such as NKG2D ligands, the same drugs also increase the expression of immunosuppressive axes, including certain inhibitory immune checkpoints, thereby facilitating cancer immune evasion (19).

## Conclusion

In conclusion, we analyzed metabolic reprogramming, cellular stress, immune response, and their relationships in HSCC. Metabolism-related genes were identified as prognostic markers and correlate with immune infiltrates in HNSCC. These results might assist researchers to understand the mechanism of the occurrence and development of HNSCC and develop effective immunotherapeutic strategies.

## Data availability statement

The datasets presented in this study can be found in online repositories. The names of the repository/repositories and accession number(s) can be found in the article/**Supplementary Material**.

## Ethics statement

The studies involving human participants were reviewed and approved by Ethics Committee of Qilu Hospital of Shandong University. The patients/participants provided their written informed consent to participate in this study.

## Author contributions

DL and HL contributed to the conception of the study; CL performed the study and wrote the manuscript; SC, WJ, WL, DW, SC, YQ, and RG helped perform the analysis with constructive discussions. All authors contributed to the article and approved the submitted version.

## Funding

This work was supported by the National Natural Science Foundation of China (No. 82071918), Science and Technology Project of Jinan City (No. 201805053), and Shandong Provincial Natural Science Foundation, China (No. ZR2020MH280).

## Acknowledgments

We thank the NanoString Technologies for their nCounter Analysis System. We also thank Dr. Yan for her patient guidance and help.

## Conflict of interest

The authors declare that the research was conducted in the absence of any commercial or financial relationships that could be construed as a potential conflict of interest.

## Publisher’s note

All claims expressed in this article are solely those of the authors and do not necessarily represent those of their affiliated organizations, or those of the publisher, the editors and the reviewers. Any product that may be evaluated in this article, or claim that may be made by its manufacturer, is not guaranteed or endorsed by the publisher.

## Abbreviations

DC, dendritic cells; ECM, extracellular matrix; ER, endoplasmic reticulum; HSCC, hypopharyngeal squamous cell carcinoma; HNSCC, head and neck squamous cell carcinomas; NK, natural killer; TCGA, The Cancer Genome Atlas; TILs, tumor infiltrating lymphocytes; TME, tumor microenvironment; OS, overall survival; SCC, squamous cell carcinoma.
