# Supplementary material for: Identify metabolism-related genes IDO1, ALDH2, NCOA2, SLC7A5, SLC3A2, LDHB, and HPRT1 as potential prognostic markers and correlate with immune infiltrates in head and neck squamous cell carcinoma
Source: Front Immunol. 2022 Aug 25;13:955614. doi: 10.3389/fimmu.2022.955614 (PMC9455275; doi:10.3389/fimmu.2022.955614)
Supplement: Supplementary file 1 [file DataSheet_1.pdf]

Table S1 Gene coverage across core metabolic themes of nCounter® Metabolic Pathways Panel.

Theme	Description	Pathways	Number of Human Genes	Number of Mouse Genes
Biosynthesis and Anabolic Pathways	The processes involved in the production of complex macromolecules by enzyme-catalyzed biosynthetic pathways. The products of these pathways are required for nearly all cellular functions, including proliferation.	Amino Acid Synthesis, Arginine Metabolism, Cell Cycle, Fatty Acid Synthesis, Glutamine Metabolism, Glycolysis, IDH1/2 Activity, Mitochondrial Respiration, Nucleotide Synthesis, Pentose Phosphate Pathway, Tryptophan/Kynurenine Metabolism, Vitamin and Cofactor Metabolism	354	348
Cell Stress	Cells are impacted by the availability of nutrients and presence of toxic compounds. Adaptive responses to the stress are required for tumorigenesis, metastasis, and immune responses.	DNA Damage Repair, Hypoxia, KEAP1/NRF2 Pathway, Reactive Oxygen Response	82	82
Nutrient Capture and Catabolic Pathways	The processes involved in the breakdown of macromolecules, scavenging of cellular materials, or import of nutrients in order to stimulate ATP production or fuel anabolic pathways.	Amino Acid Transporters, Autophagy, Endocytosis, Fatty Acid Oxidation, Glucose Transport, Lysosomal Degradation, Nucleotide Salvage	161	159
Metabolic Signaling	The pathways that are commonly disrupted in cancer cells or altered in immune cells that impact metabolic function. In the context of cancer, mutations allow these regulated signaling pathways to allow for metabolic change enabling tumorigenesis.	AMPK, mTOR, MAPK, Myc, NF-kB, p53 Pathway, PI3K, TCR and Costimulatory Signaling, TLR Signaling	237	235
Transcriptional Regulation	Processes involved in the alteration of epigenetic and transcriptional activity of the cell that enables sustained metabolic reprogramming. This reprogramming allows for tumorigenesis and underlies stable changes in immune cell phenotype.	Epigenetic Regulation, Transcriptional Regulation	77	69

Table S2 Selected housekeeper genes for normalization.

Gene Name	Gene	Order selected by geNorm	SD after normalization
EDC3-mRNA	NM_001142443.1:1024	1	0.234
POLR2A-mRNA	NM_000937.2:3775	2	0.279
COG7-mRNA	NM_153603.3:1492	3	0.319
SDHA-mRNA	NM_004168.3:342	4	0.323
NRDE2-mRNA	NM_017970.3:3233	5	0.341
FCF1-mRNA	NM_015962.4:1022	6	0.401
AGK-mRNA	NM_018238.3:816	7	0.364
MRPS5-mRNA	NM_031902.3:390	8	0.403
DHX16-mRNA	NM_001164239.1:2490	9	0.399
DNAJC14-mRNA	NM_032364.5:1166	10	0.38
TBC1D10B-mRNA	NM_015527.3:2915	11	0.401
SAP130-mRNA	NM_024545.3:3090	12	0.446
TLK2-mRNA	XM_011524223.1:383	13	0.471
STK11IP-mRNA	NM_052902.2:565	14	0.505
TBP-mRNA	NM_001172085.1:587	15	0.514
USP39-mRNA	NM_001256725.1:806	discarded	0.767
OAZ1-mRNA	NM_004152.2:313	discarded	0.792
G6PD-mRNA	NM_000402.4:923	discarded	0.875
UBB-mRNA	NM_018955.3:1052	discarded	1
ABCF1-mRNA	NM_001090.2:857	discarded	0.598

Table S3 mRNA normalization summary of samples.

	mRNA.normalization.factors	HK.MSE
H01166ca	0.769476963	0.259346
H01166m	-1.00153961	0.507071
H01184ca	0.70807074	0.547514
H01184m	-1.15182492	0.427918
H01215ca	-0.46183283	0.427621
H01215m	-2.22956612	0.242699
H01278ca	-0.18614079	0.069782
H01278m	-0.98438077	0.159465
H01346ca	0.068790222	0.238649
H01346m	-0.89711135	0.609704
H01365ca	0.282494597	0.087934
H01365m	0.209975806	0.119805
H01186ca	-1.00118969	0.271501
H01186m	0.455831977	0.128761
H01216m	0.951287641	0.228739
H01271ca	0.851576425	0.17497
H01271m	-0.2560087	0.223001
H01384ca	1.373016841	0.57622
H01384m	0.207630097	0.055594
H01389ca	1.399576888	0.184764
H01389m	0.529590925	0.274384
H01406ca	0.824294275	0.25412
H01406m	-0.46201861	0.160228

Figure S1

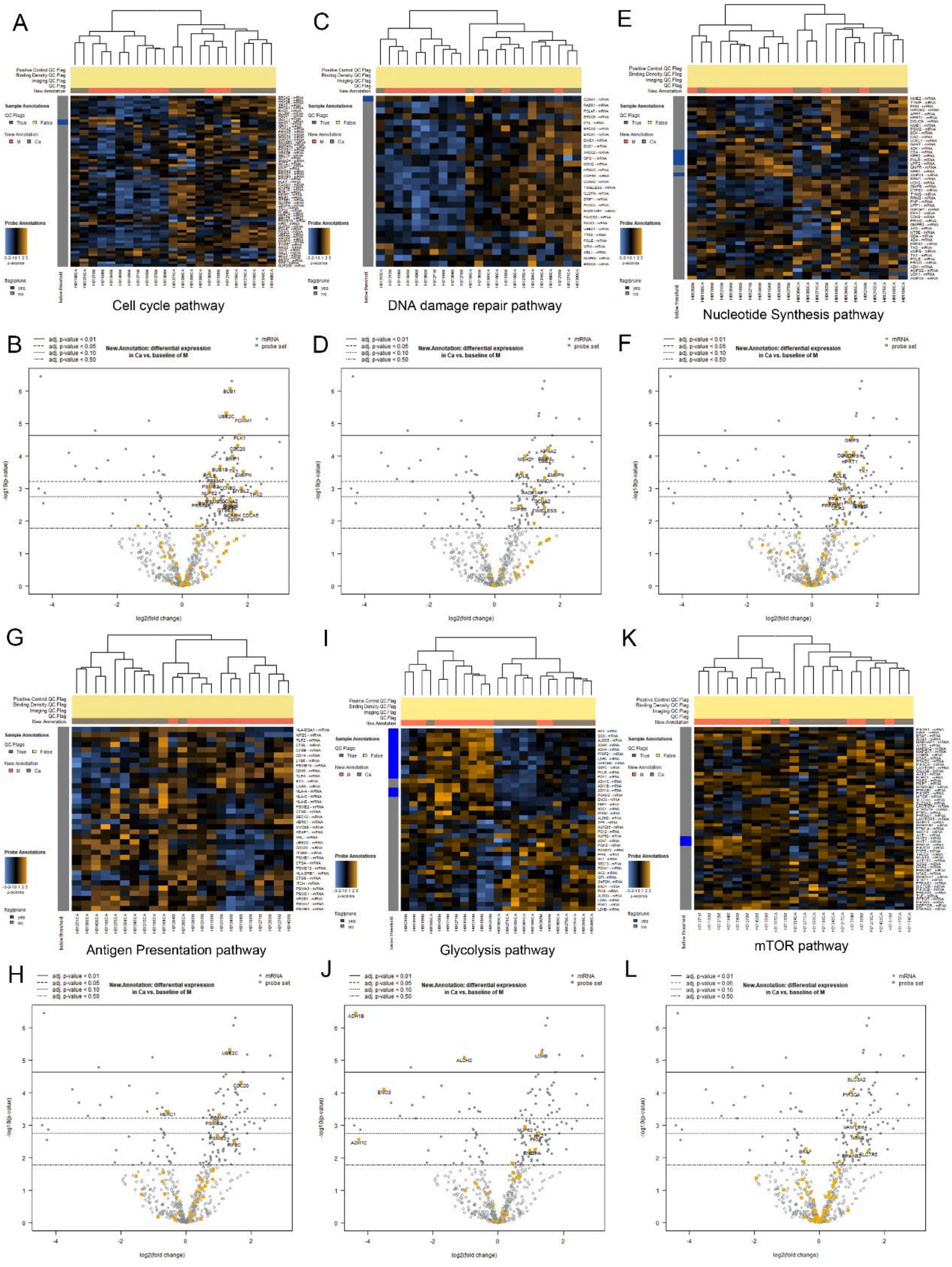
A
Cell cycle pathway
B
C
DNA damage repair pathway
D
E
Nucleotide Synthesis pathway
F
G
Antigen Presentation pathway
H
I
Glycolysis pathway
J
K
mTOR pathway
L

Figure S1 Analysis of significantly up-regulated signaling pathways and related genes in nCounter® Metabolic Pathways Panel between HSCC tissues and adjacent normal tissues.

A Heatmap of Cell Cycle pathway. **B** Volcano plot of Cell Cycle pathway. **C** Heatmap of DNA Damage Repair pathway. **D** Volcano Plot of DNA Damage Repair pathway. **E** Heatmap of Nucleotide Synthesis pathway. **F** Volcano plot of Nucleotide Synthesis pathway. **G** Heatmap of Antigen Presentation pathway. **H** Volcano plot of Antigen Presentation pathway. **I** Heatmap of Glycolysis pathway. **J** Volcano plot of Glycolysis pathway. **K** Heatmap of mTOR pathway. **L** Volcano plot of mTOR pathway.

Figure S2

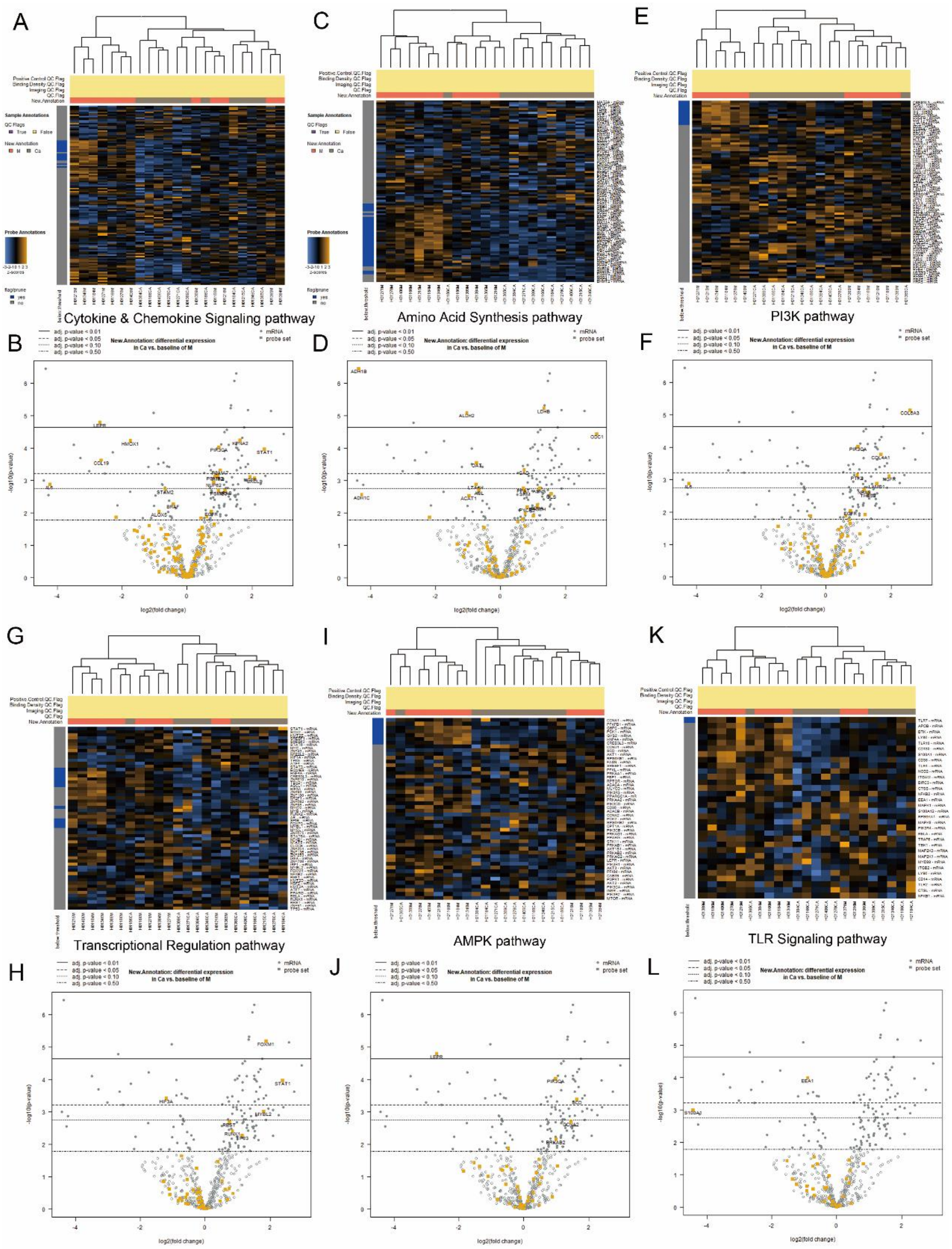
A
Cytokine & Chemokine Signaling pathway
B
C
Amino Acid Synthesis pathway
D
E
PI3K pathway
F
G
Transcriptional Regulation pathway
H
I
AMPK pathway
J
K
TLR Signaling pathway
L
adj. p-value < 0.01
adj. p-value < 0.05
adj. p-value < 0.10
adj. p-value < 0.50
New.Annotation: differential expression in Ca vs. baseline of M
mRNA
probe set
log2(fold change)
-log10(p-value)

Figure S2 Analysis of Significantly down-regulated signaling pathways and related genes in nCounter® Metabolic Pathways Panel between HSCC tissues and adjacent normal tissues.

A Heatmap of Cytokine & Chemokine Signaling pathway. **B** Volcano plot of Cytokine & Chemokine Signaling pathway. **C** Heatmap of Amino Acid Synthesis pathway. **D** Volcano Plot of Amino Acid Synthesis pathway. **E** Heatmap of PI3K pathway. **F** Volcano plot of PI3K pathway. **G** Heatmap of Transcriptional Regulation pathway. **H** Volcano plot of Transcriptional Regulation pathway. **I** Heatmap of AMPK pathway. **J** Volcano plot of AMPK pathway. **K** Heatmap of TLR Signaling pathway. **L** Volcano plot of TLR Signaling pathway.

Figure S3

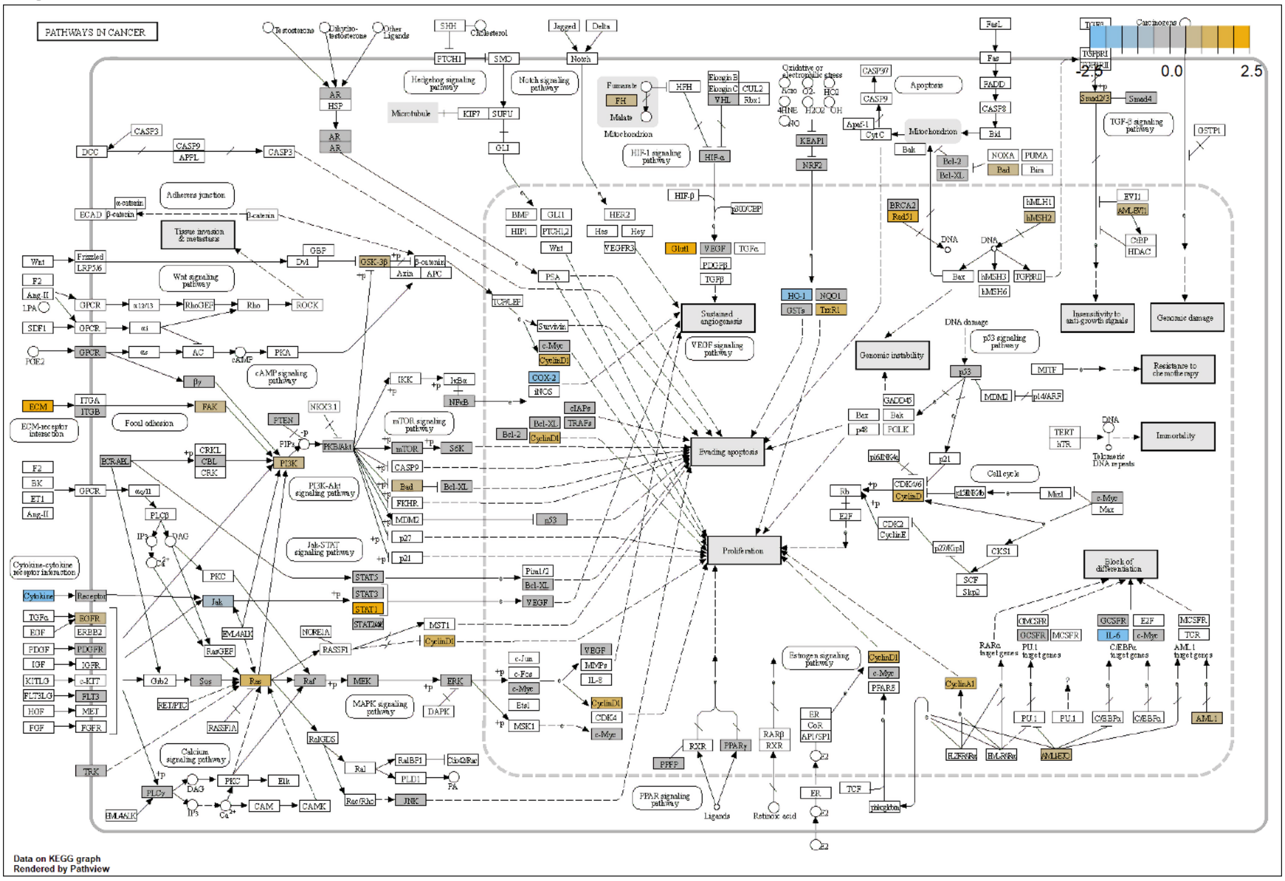

Figure S3 Analysis of altered gene in HSCC in cancer pathway.

For cancer KEGG pathways, genes within panels are mapped to pathways, and differential expression information is overlaid on protein-based KEGG pathway images.
